# An exploration of registered dietitian accreditation system development in China

**DOI:** 10.1186/s12909-022-03802-z

**Published:** 2022-12-07

**Authors:** Zhang Yajie, Wang Xiaoli, Liu Ya, Shen Xiuhua, Xiao Rong, Zhu Huilian, Zeng Guo, Cai Wei, Ma Aiguo, Yang Yuexin

**Affiliations:** 1 Xinhua Hospital, School of Medicine, Shanghai Jiao Tong University, 200092 Shanghai, China; 2 Shanghai Institute for Pediatric Research, Shanghai, China; 3 Chinese Nutrition Society, 100053 Beijing, China; 4 School of Public Health, Jilin University, Changchun, China; 5 Department of Nutrition, School of Medicine, Shanghai Jiao Tong University, 200092 Shanghai, China; 6 School of Public Health, Capital Medical University, Beijing, China; 7 School of Public Health, Sun Yat-Sen University, Guangzhou, China; 8 West China School of Public Health, Chengdu, China; 9 Department of Pediatric Surgery, Xinhua Hospital, School of Medicine, Shanghai Jiao Tong University, Shanghai, China; 10 The College of Public Health, Qingdao University, 266021 Qingdao, China; 11 Chinese Registered Dietitian Commission, Chinese Nutrition Society, 100053 Guangbodasha, Beijing, China

**Keywords:** Dietetic education, Dietetic professional, Registered dietitian, Dietetic accreditation

## Abstract

The registered dietitian system started late in China, and since 2014 a working committee of registered dietitians has been formed by the Chinese Nutrition Society to establish a national accreditation system in accordance with the international standard academic and internship requirements. The data from the certification examinations and registrations were collected and analyzed. 8,328 registered dietitians were included in the study, with 5,673 registered dietitians (RD) and 2,655 registered dietetic technicians (DTR). Among them, 45.27% of RDs have master degree or above, and 66.44% of DTRs have bachelor or above. 53.25% RDs work in medical institutions, 14.77% in colleges and universities, and 14.56% in enterprises; 48.21% DTRs work in the medical institutions, 30.28% in enterprises, and 7.34% in the community. Although six years of work has initially established a foundation of standardized registered dietitian accreditation system, the problems of limited resources for academic education and the lack of legislative protection for the profession remain prominent.

**Supplementary Information:**

The online version contains supplementary material available at 10.1186/s12909-022-03802-z.

## Background

In the early 20 century, the modern registered dietitian accreditation system originated and shaped the education and practice of dietitians as a profession of medical team in western countries.[1] In China, “Physician Specialized in Diet” as a profession was recorded as early as 12th century BC (Zhou Dynasty) in *Rites of Zhou*.[2, 3] Modern dietetic programs with a bachelor’s degree had been first established in 1921 with the Peking Union Hospital, which was a similar dietitian training program as it was in the United States.[4, 5] However, all of the programs had been suspended in 1952.[5] It was not until 1985 that degree education of nutrition has been restored as a five-year program in medical school of four universities.[6] Although the training period is the same as that of a physician, dietitians are classified as medical technicians with limited pay and career advancement.[4, 5] As a result, the number of well-trained dietitian is far from sufficiency that most professionals who worked in hospitals’ nutrition departments were physicians and nurses until 2004.[5].

In 2004, the Ministry of Education of China established a four-year undergraduate program in nutrition, while Shanghai Jiao Tong University was the first school to offer this major.[4, 7] To build a team of dietitians with guaranteed competencies and number, as the only nationwide nutritional science academic group in China, the Chinese Nutrition Society (the CNS) began to design a registered dietitian accreditation system in 2014.[8] In 2016, the government started to *promote de-regulation and improve service*, thus the government-led vocational qualification certification was gradually transferred to professional level evaluation, with the participation of professional institutions and academic associations.[9] In this context, the CNS assembled a particular committee to set sail of the dietetic certifications in accordance with international models.[8].

In 2015, the CNS and Shanghai Jiao Tong University jointly initiated the training and certification of registered dietitians based on international standards. In May of 2016, the first registered dietitian examination (pilot) was successfully held in Shanghai.[8, 10].

It has now been six years since its inception. This article reviews the highlights of the registered dietitian accreditation system in China, including its organization, regulatory policies, certification requirements, development of and performance on the certification examination, registration, and continuing education. A brief analyses and prospect are also discussed.

## The RD/DTR accreditation system in China

### Organization

The CNS is the organization responsible for the accreditation system of registered dietitians in China. Founded in 1945, the CNS is a national non-profit academic organization dedicated to bringing together nutrition scientists and professionals to advance the science of nutrition and to support the dissemination and application of nutrition to improve human well-being and prevent disease in China and the world. Currently, the CNS has over 35,000 members in 31 provinces across China, including academic professionals, nutritionists, registered dietitians, health professionals, educators, and students interested in pursuing a career in the field of nutrition.[11].

In December 2016, the Registered Dietitian Committee (RDC) was formally established by the CNS. The committee recruited a total of 45 representatives of dietetics and nutrition experts from universities, hospitals, and the Centers for Disease Control. The Committee consists of two branches, one for degree education and examinations, and the other for registration, supervision and regulation. The Education and Training Department of the Secretariat of the CNS is responsible for day-to-day management.[12].

The committee is also responsible for didactic and internship program management. Institutions may apply to the committee for program accreditation, and the committee evaluates and accredits these applications. The purpose of accreditation and supervision is to standardize curriculum and internship content. At present, 45 degree programs have met the requirements of curriculum and faculty, including 12 “double first-class universities” in 27 provinces, autonomous regions, and municipalities across the country. Among the approved institutions, 41 can train both registered dietitians (RDs) and dietetic technicians, registered (DTRs), while four can train only DTRs (Appendix 1). By now, 115 accredited internship programs meet the criteria including hospitals, community and enterprises.[13, 14].

### Regulation

The committee draws on the advanced experience of the International Confederation of Dietetic Associations (ICDA), the United States, Japan, as well as other countries and regions. In establishing minimum standards for dietetics’ education in China, the CNS sought guidance from ICDA documents including those describing ethical conduct, academic degree, competency standards, dietetics internships or professional placements. The minimum requirements are a bachelor’s degree or higher in nutrition and related fields and have completed at least 500 h of practical experience. Considering local facts, it has established a series of provisions and regulations of the registered dietitian accreditation system,[15] including *Interim Provisions on the Level Evaluation System of Registered Dietitians*,[16] *Implementation of the Level Evaluation Examination of Registered Dietitians*,[17] *Interim Measures for the Management of Continuing Education of Registered Dietitians*,[18] *Regulations of Didactic and Internship Programs Accreditation*, and *Professional Code of Ethics and Competency Standards*.[15].

### Pathway to be an RD/DTR

According to the provisional and regulatory documents released, the credentialing process to be an RD or a DTR includes the corresponding curricula, internship, examination, registration and re-registration which were summarized in Table 1. The RD’s role is to use evidence-based nutrition science to engage in diet management, nutrition support, medical nutrition therapy, and nutrition counseling for individuals or groups; DTRs typically engage in diet management and nutrition counseling under the supervision of a registered dietitian. [16]

**Table 1:** The Criteria for RD and DTR Registration

	Registered Dietitian	Registered Dietetic Technicians
Academic Coursework*	1) Complete a minimum of a bachelor degree in dietetics and nutrition majors	1) Complete a minimum of an associate degree**** in dietetics and nutrition majors or a bachelor degree in any majors
	2) Complete a series of required courses for Registered Dietitians (see Appendix 1) and acquire corresponding academic credits (800 h)	2) Complete a series of required courses for Registered Dietetic Technicians (see Appendix 1) and acquire corresponding academic credits (432 h)
Internship*	Work in nutrition and nutrition-related fields or complete the dietetic internship for at least one year	Work in nutrition and nutrition-related fields or complete the dietetic technical internship for at least one year
Credential Examination**	Pass the exam	Pass the exam
Registration (re-registration*)	Apply to the Committee	Apply to the Committee
Continuing Education***	50 credits (400 h) in five years	30 credits (240 h) in five years

Abbreviations: RD, registered dietitian; DTR, dietetic technicians, registered

Notes: * *Interim Provisions on the Level Evaluation System of Registered Dietitians*; ** *Implementation of the Level Evaluation Examination of Registered Dietitians*; *** *Interim Measures for the Management of Continuing Education of Registered Dietitians*; **** *An associate degree refers to a three-year college degree*

### Academic coursework

The training of RD and DTR in China is based on degree education. At least a bachelor degree is required to obtain an RD credential, and an associate degree is required for DTR. The core curriculum of registered dietitian is 800 h, and the registered dietetic technician is 432 h, including ten modules of general medicine, fundamental nutrition, food science and food safety, foodservice and management, community nutrition, human nutrition, clinical nutrition, nutrition education, environment and health and comprehensive practice. The course requirements for RD and DTR differ in depth and credits that the detailed curricula are listed in Appendix 2.

### Internship

For a competent RD/DTR, supervised practice is essential. The internship program needs to include three directions of practice including clinical nutrition, public nutrition and food nutrition. The internship spots of clinical nutrition direction is in hospitals, clinics, and maternal and child health institutions; the public nutrition direction is in CDC, nursing homes, schools and communities; the food nutrition direction is in food processing, catering and other related enterprises. The full internship cumulative time of either type of the programs shall not be less than one year.[13].

### Examination

The ones who completed the didactic and internship programs would be eligible to sit for the registration exam.

The content of the RD examination is divided into four modules that (1) Individual and Group Nutrition Management accounts for 40% of the total, (2) Food and Nutrition, (3) Public Nutrition and Nutrition Education, and (4) Foodservice Management each accounts for 20%; the content of the DTR examination has three modules that (1) Food Science and Foodservice Management accounts for 50% of the total, and (2) Individual and Group Nutrition Management and (3) Public Nutrition and Nutrition Education each accounts for 25%. The grades would be announced three months after the exams.[17].

### Registration

The candidates with a passing grade need to apply to the Committee for registration. Registration is valid for five years. One must apply to the Committee for re-registration three months before it expires. Candidates who are re-registered must meet the following criteria:[1] complete the minimum credit requirements for continuing education; [2] comply with the Code of Ethics; [3] the employers have no objection against the applicants in obtaining certificates of RD or DTR.[16].

### Continuing education

To ensure the qualification’s activeness, RD must obtain at least 50 credits, while DTR must obtain at least 30 credits in continuing education practices in 5 years after registered.[18].

The following educational activities account for claiming credits for the Continuing Education record: [1] participate in accredited Continuing Education courses for Registered Dietitian; [2] enroll in a full-time professional study for at least one month; [3] participate in academic conferences, professional workshops, and related training; [4] publish journal articles, presentations, or books; [5] accredited continuing education programs by either national, provincial or municipal government departments; conduct health promotion articles and books via newspaper, magazine, or selected platforms; attend academic conferences, and submit abstracts; others including participation in academic material compiling, teaching, and attaining a degree.[18].

## Review on the examinations

Since 2016, national registered dietitian examinations have been organized successfully for five consecutive years, including four RD exams and two DTR exams. The credential examination sites are set up in five different cities across the country with the assistance of universities.[19, 20] Since 2020, CNS has commissioned professional test agencies and the number of test sites has been increased to be in 15 cities.[21].

The pass rates were listed in Table 2. The first nationwide test was free of examination fees,[19] the Chinese Nutrition Society bears all costs, and the paper-based test RD 280 yuan/person/time,[22] DTR 200 yuan/person/time,[20] computer-based RD 380 yuan/person/time, DTR 300 yuan/person/time.[21].

**Table 2:** Statistics of RD and DTR Examinations

Level	Year	Examinee	Pass	% Pass
RD	2016	193	62	32.12%
	2017	1414	509	36.00%
	2018	2602	786	30.21%
	2019	3443	1074	31.19%
DTR	2017	1053	697	66.68%
	2018	1562	856	54.80%

Abbreviations: RD, registered dietitian; DTR, dietetic technicians, registered

Table 3 listed the pass rate of test attendees with different degree levels from 2017 to 2019. In terms of RD, the higher the degree of candidates, the higher the pass rate. The pass rate of doctoral students with a higher pass rate is more than 20%, different from undergraduates with a lower pass rate. In terms of DTR, the pass rates for associates and bachelors are similar, and the pass rates for masters and doctors are similar, with a difference of about 20%.

**Table 3:** Pass Rate of RD and DTR Examinations with Different Degree Levels

Level	Degree Level	2017	2018	2019
RD	Bachelor’s	40%	30%	32.7%
	Master’s	51.9%	41.9%	42.4%
	Doctoral	62.5%	58.3%	53.8%
DTR	Associate’s	64.16%	50.69%	-
	Bachelor’s	62.39%	54.64%	-
	Master’s	82.25%	70.71%	-
	Doctoral	83.33%	57.14%	-

Abbreviations: RD, registered dietitian; DTR, dietetic technicians, registered

Note: Absentees excluded.

## Analyses on current registered

There are currently 5673 RDs (68.12%) and 2655 DTRs (31.88%), of which 80.58% are females, and 19.42% are males.

As 2017–2019 was the dietitian system’s transition period, those who meet the academic requirements and have worked for more than 15 years in the field may apply for an exemption.[23] Also, an increasing number of overseas registered dietitians are returning to work in China. Chinese with foreign RD/DTR credentials from the United States, Canada, Australia, New Zealand, and Japan are also eligible to apply for an exemption.[24] The composition of credentials, including test passed, test waived and overseas exempted, and the geographical distribution are listed in Table 4. As shown, the geographical distribution of RD/DTR is extremely uneven, more than 63% of the total amount of RD/DTR are concentrated in 10 well-developed provinces and cities such as Guangdong, Beijing and Shanghai.

**Table 4:** Selected Characteristics of Current Registered in China

Variable	n = 8328 (%)
**Level**	
RD	5673 (68.12%)
DTR	2655 (31.88%)
**Gender**	
Female	6711 (80.58%)
Male	1617 (19.42%)
**Composition of credentials**	
Test passed*	3897 (46.79%)
Test waived**	4375 (52.53%)
Overseas	56 (0.67%)
**Geographical distribution (top 10 and others)**	
Guangdong Province (South)	987 (11.85%)
Beijing (North)	874 (10.49%)
Shanghai (East)	727 (8.73%)
Zhejiang Province (East)	554 (6.65%)
Jiangsu Province (East)	533 (6.40%)
Sichuan Province (West)	348 (4.18%)
Shandong Province (East)	336 (4.03%)
Shanxi Province (Northwest)	335 (4.02%)
Jilin Province (Northeast)	313 (3.76%)
Liaoning Province (Northeast)	289 (3.47%)
Others	3032 (36.41%)

Abbreviations: RD, registered dietitian; DTR, dietetic technicians, registered

Notes: *Those who have a bachelor’s degree or higher and have worked as a dietitian for more than 15 years can apply to be exempted from the Registered Dietitian Examination, and those who pass the qualification audit can obtain the certificate directly; **Holders of registered dietitian certificates in the United States, Canada, Japan, New Zealand and Australia can apply for exemption from the registered dietitian examination and receive the certificate directly if they pass the qualification audit

### Age

**Fig. 1:**
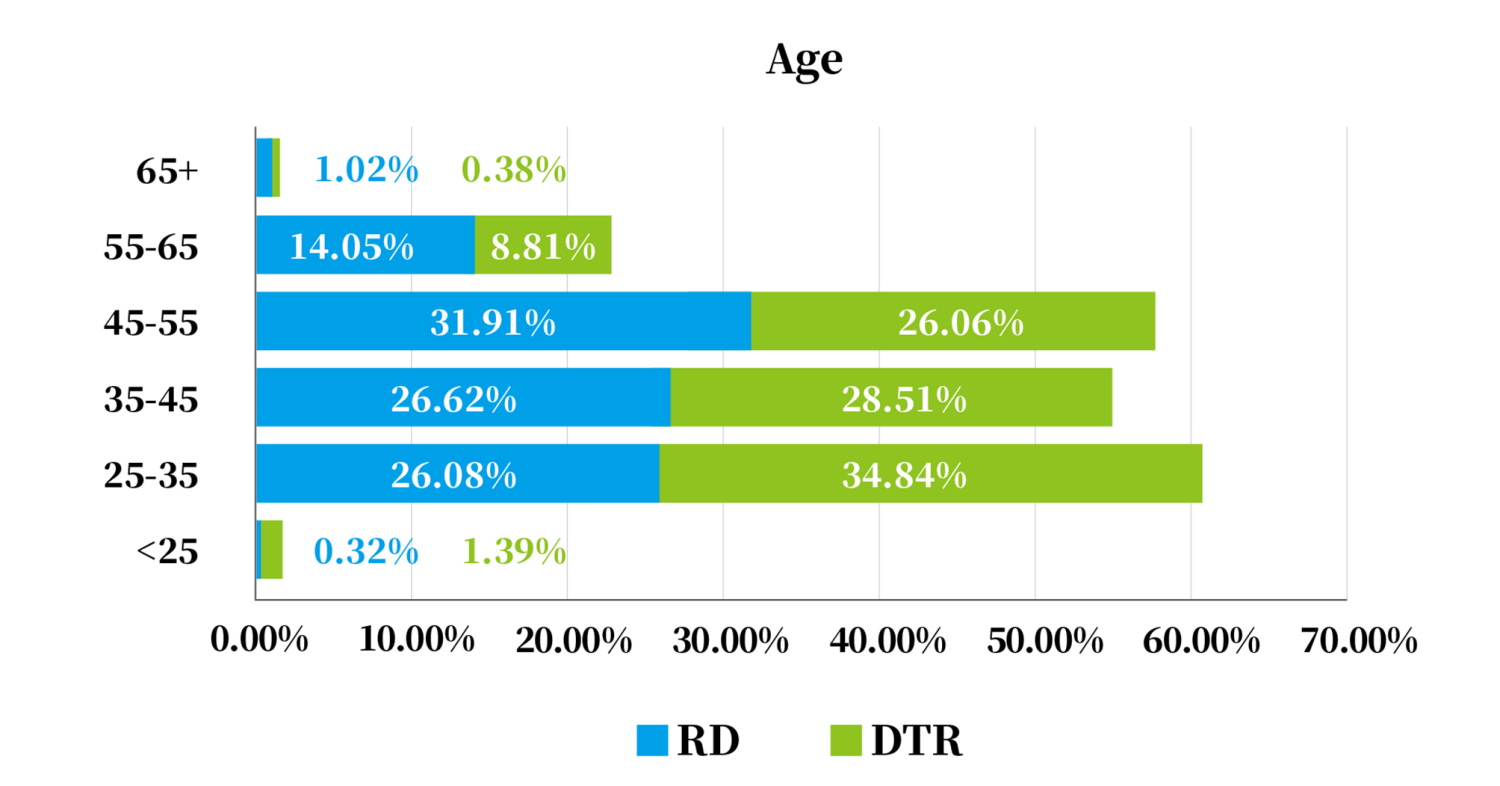
The age structure of RDs and DTRs. Abbreviations: RD, registered dietitian; DTR, dietetic technicians, registered

The age ranges from 24–105 years old. As shown in Fig. 1, RDs accounted for the highest proportion of 45–54 years old while the proportion of DTRs in 25–34 years old was the highest. The proportion of both RDs and DTRs aged less than 25 years old and over 65 years old is the smallest, accounting for less than 2% of the total. The percentage of RDs aged 35 years or older is 73.6%, while it of DTRs is 63.76%.

### Educational background

**Fig. 2:**
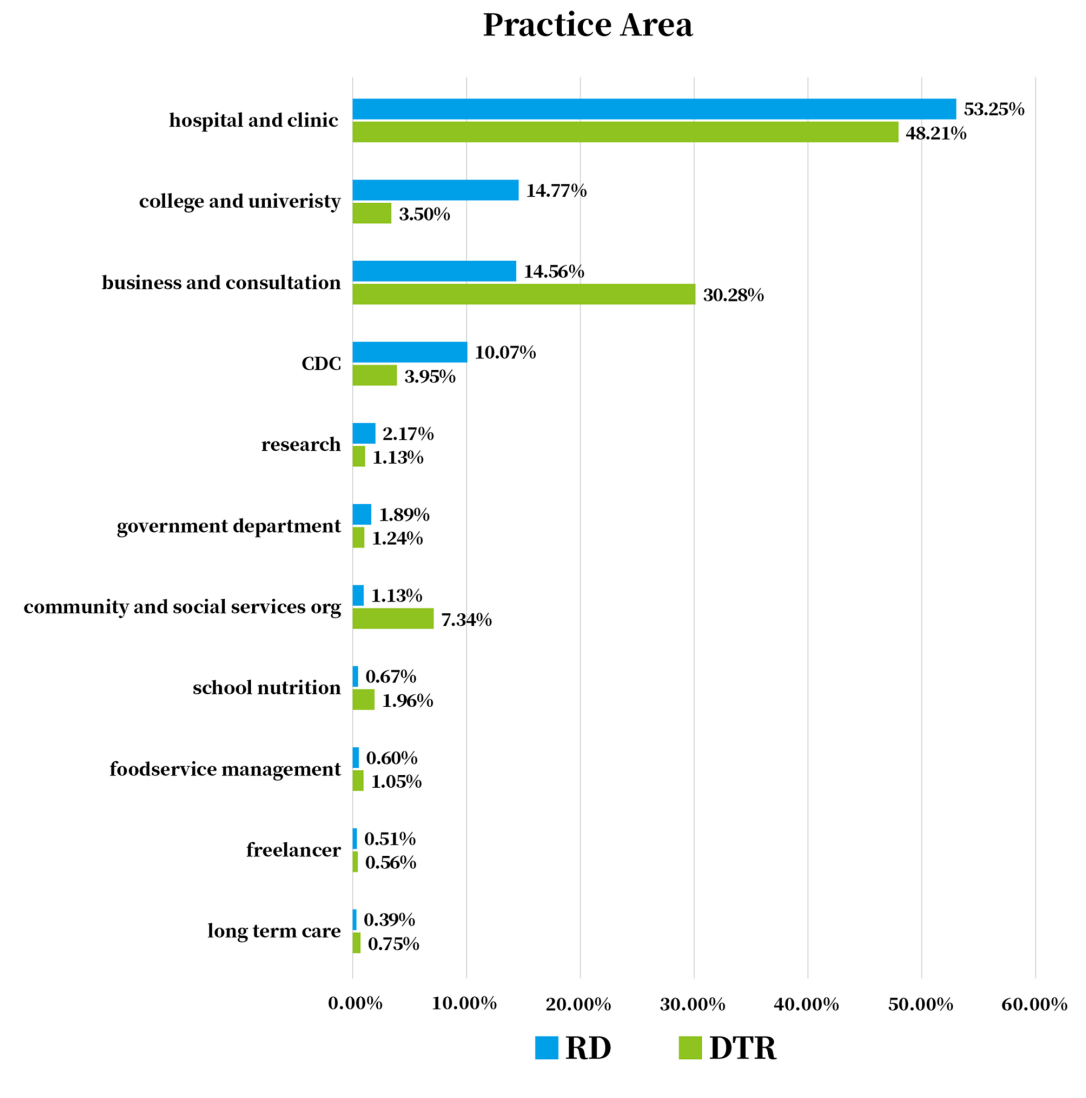
The education background of RDs and DTRs. Abbreviations: RD, registered dietitian; DTR, dietetic technicians, registered

 According to Fig. 2, the overall educational level of RDs is higher than that of DTRs. Bachelor’s degree accounts for a large proportion of the qualifications of RDs and DTRs, reaching 53.39% and 54.95%, respectively. In terms of master’s degree and doctoral degree, the proportion of highly educated RDs was 45.27%, which was significantly higher than 11.49% of DTRs. The gap between RDs and DTRs in master’s degree is 20.61%, while that in doctor’s degree is 13.17%. It is worth noting that even 0.79% of DTRs have a doctoral degree, while 1.34% of registered dietitians have an associate degree.

### Practice areas

**Fig. 3:**
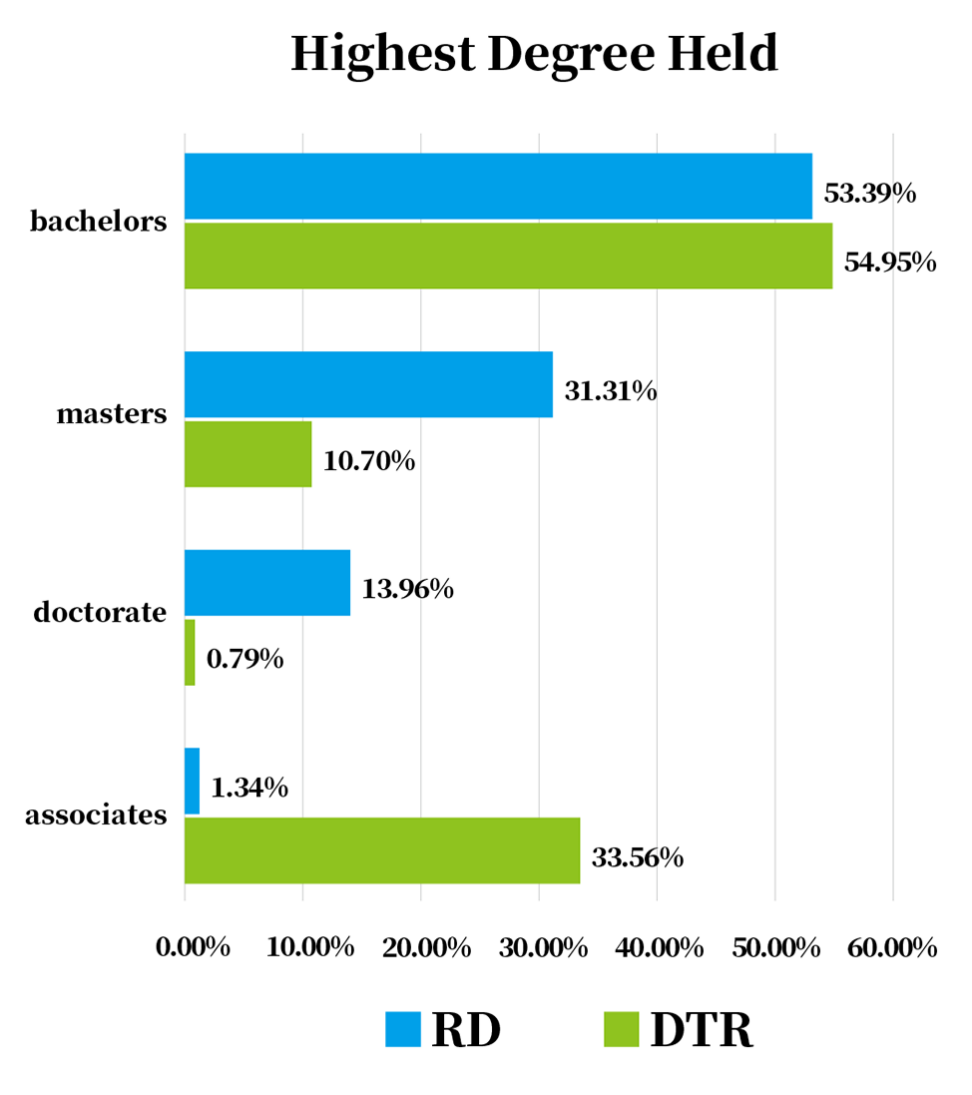
Practice Area of RDs and DTRs. Abbreviations: CDC, Centers for Disease Control and Prevention; RD, registered dietitian; DTR, dietetic technicians, registered

 As shown in Fig. 3, both RDs and DTRs accounted for the highest proportion of working in hospitals and clinics, reaching 53.25% and 48.21%, respectively. By comparing the occupational distribution of RDs and DTRs, it is not difficult to see that the proportion of RDs from enterprises, educational institutions, and Centers for Disease Control and Prevention is more than 10%, while DTRs are mainly from business and community. Other practice areas such as research institutions, government departments, school nutrition, food service management, and long-term care are relatively low in proportions.

## Discussion and conclusion

 China is the most populous country in the world. With economic development and improved awareness of nutrition, there have been enormous demands for competent dietitians. Qualified personnel are essential to provide nutrition care to the public were specified in *the National Nutrition Plan 2017–2030* issued by the government in 2017. For example, it stipulates that “strengthen the establishment of clinical nutrition departments so that the ratio of dietitians to hospital beds shall reach 1:150.”[25, 26] However, the current ratio for hospital dietitians to patients is 1:807, not including community hospitals.[27] Both the number and quality of dietitians in China are far from meeting the needs. Therefore, it is necessary and urgent to build a team of qualified and skilled dietitian with sufficient amount.

 A six year review of the development of the registered dietitian accreditation system in China shows that great efforts have been made and significant progress have been achieved. The system has reached or approached the international standards in terms of certification standards, curriculum design, internship requirements, examination content,[28, 29] and pass rates.[30, 31] Given the system implementation is only in the primary stage, the amount of competent dietitians is still inadequate. There are currently only 0.67 RD/DTR per 100,000 people in China with uneven geographical distribution, which is much lower than around 194 in Japan[32] and 33 in the United States.[33].

 At present, the construction and development of China’s registered dietitian team is also facing some obstacles. The first is the lack of institutional protection. Due to unclear career positioning and the role of dietitian has been relegated to technician, doctors and nurses undertake part of the work of dietitians, and thus the employment of dietitians is not guaranteed. Second, the resources for academic education in nutrition is insufficient. As of the end of 2021, China has only 45 colleges and universities with four-year dietetic programs, with an annual enrollment of about 1,500 students. The United States currently has more than 600 accredited registered dietitian academic education and internship programs; Japan has 152 universities that train management dietitians and 167 schools that train dietitians.[34] As seen in age distribution, the percentage of RDs aged 35 years or older is 73.6%, while it of DTRs is 63.76%. It might imply that there is a lack of fresh blood in the talent pool. The problems in lack of institutional protection and educational resources can also be seen in countries with newly develop registered dietitian accreditation system such as Vietnam.(35) Furthermore, because of the uneven distribution of medical resources, the dietitian team is even more geographically vast differences that China’s registered dietitians are mainly concentrated in developed coastal cities such as Beijing, Shanghai, Guangzhou, Jiangsu and Zhejiang, and there is a serious shortage of dietitians in central and western China and rural areas. Also, the competencies have not been fully adapted to the dietetic programs that there is still gap between the document provisions and actual implementation. The low passing rates might also imply that the academic education and internship are not strictly in accordance with the training objectives, and more supervision and management are needed to ensure that the content of the exam syllabus and professional competence requirements are incorporated into the regular training and that the students try their best to master the knowledge and skills. Hence, it would be a long way to keep the system effective. More data and research on how the competencies are implemented currently and what would be the effect in career development of RD/DTR to improve the system next step.

 Based on the consideration above, in the forthcoming years, the following aspects should be focused: (1) Promote academic education and supervised practice in dietetics, strengthen the discipline construction of dietetics majors, expand the number of qualified programs; (2) Conduct follow-up surveys of certified dietitians entering the workplace to understand the actual knowledge and skill needs for the profession to better meet market needs; (3) Create a favorable and supportive policy environment in the health industry, encourage more highly educated talents to engage in health and nutrition field to protect the health, safety, and welfare of the public.

## Electronic supplementary material

Below is the link to the electronic supplementary material.

Supplementary Material 1

## Data Availability

The datasets used and/or analyzed during the current study are available from the corresponding author on reasonable request.

## List of abbreviations

CNS: Chinese Nutrition Society (CNS)
CDC: Centers for Disease Control and Prevention
RD: Registered dietitian
DTR: Dietetic technicians, registered
