# Supplementary material for: An exploration of registered dietitian accreditation system development in China
Source: BMC Med Educ. 2022 Dec 7;22:846. doi: 10.1186/s12909-022-03802-z (PMC9727847; doi:10.1186/s12909-022-03802-z)
Supplement: Supplementary file 1 — Supplementary Material 1 [file 12909_2022_3802_MOESM1_ESM.docx]

# Appendix

# *Appendix 1 – List of institutions of accredited didactic programs*

| **Region** | **Province** | **University/College** |
| --- | --- | --- |
| Northeast China | Jilin | Jilin University |
|  |  | Baicheng Medical College |
|  | Heilongjiang | Harbin Medical University |
|  | Liaoning | China Medical University |
| Northwest China | Shanxi | Shanxi University of Chinese Medicine |
|  |  | Xi'an Jiaotong University |
|  | Xinjiang | Xinjiang Medical University |
|  | Ningxia | Ningxia Medical University |
| North China | Beijing | Peking University |
|  |  | Capital Medical University |
|  |  | Beijing Union University |
|  | Tianjin | Tianjin Medical University |
|  |  | Tianjin University of Science &Technology |
|  | Hebei | Hebei Medical University |
|  | Shanxi | Shanxi Medical University |
| Central China | Hubei | Huazhong University of Science and Technology |
|  | Hunan | Central South University |
|  | Henan | Zhengzhou University |
| East China | Shandong | Qingdao University |
|  |  | Shandong University |
|  | Jiangsu | Southeast University |
|  |  | Nanjing Medical University |
|  |  | Nanjing University of Chinese Medicine |
|  |  | Yangzhou University |
|  |  | Suzhou Vocational Health College |
|  | Anhui | Anhui Medical University |
|  |  | Bengbu Medical College |
|  | Zhejiang | Zhejiang University |
|  |  | Wenzhou Medical University |
|  |  | Ningbo College of Health Sciences |
|  | Fujian | Fujian Medical University |
|  | Jiangxi | Nanchang University |
|  | Shanghai | Shanghai Jiaotong University |
|  |  | Fudan University |
|  |  | Shanghai University of Traditional Chinese Medicine |
|  |  | Shanghai University of Medicine & Health Sciences |
| South China | Guangxi | Guangxi Medical University |
|  |  | Guilin Medical University |
|  | Guangdong | Sun Yat-sen University |
|  | Hainan | Hainan Medical University |
| Southwest China | Sichuan | Sichuan University |
|  |  | Chengdu University of T.C.M |
|  | Yunnan | Kunming Medical University |
|  | Guizhou | Guizhou Medical University |
|  | Chongqing | Southwest University |

# *Appendix 2 – Dietetic curriculum*

| **Modules** | **Registered Dietitian** | | **Registered Dietetic Technicians** | |
| --- | --- | --- | --- | --- |
|  | **Courses** | **Credits** | **Courses** | **Credits** |
| General Medicine | Anatomy, Biochemistry, Physiology, Microbiology, Immunology | 10 | Biochemistry, Physiology | 1 |
| Fundamental Nutrition | Introduction to Nutrition, Nutrition Foundation | 4 | Introduction to Nutrition, Nutrition Foundation | 3 |
| Food Science and Food Safety | Food Science, Food Analysis, Food Processing, Food Safety | 6 | Food Science, Food Safety | 5 |
| Foodservice and Management | Culinary Science, Diet Management and Design, Recipe Preparation, Catering Management and Practice | 6 | Culinary Science, Diet Management and Design, Recipe Preparation, Catering Management and Practice | 10 |
| Community Nutrition | Dietary Nutrient Reference Intake, Dietary Guidelines, Nutrition Survey and Evaluation, Food, Health and Dietary Patterns (including Vegetarian), Community Nutrition and Chronic Disease Management, Health Management | 5 | Dietary Nutrient Reference Intake, Dietary Guidelines, Nutrition Survey and Evaluation, Food, Health and Dietary Patterns (including Vegetarian), Community Nutrition and Chronic Disease Management, Health Management | 3 |
| Human Nutrition | Nutrition of People of Different Ages (Different Physiological Conditions), Nutrition of People of Different Occupations and Environment, Sports Nutrition | 4 | Nutrition of People of Different Ages (Different Physiological Conditions), Nutrition of People of Different Occupations and Environment, Sports Nutrition | 3 |
| Clinical Nutrition | Introduction to Clinical Nutrition, Intestinal and Parenteral Nutrition, Medical Nutrition Therapy | 7 | Introduction to Clinical Nutrition | 1 |
| Nutrition Education | Psychology, Nutrition Counseling and Education, Nutrition Counseling and Educational Practice | 4 | Nutrition Counseling and Education, Nutrition Counseling and Educational Practice | 1 |
| Environment and Health | Introduction to Environment and Health, Epidemiology and Health Statistics | 3 |  | 1 |
| Dietetic Internship |  | 1 |  |  |
| Total credits |  | 50 |  | 27 |
| Total hours (16 hours per credit) |  | 800 |  | 432 |

# *Appendix 3 – Timeline: Milestones of Chinese Registered Dietitian*

|  | **TIMELINE: Milestones of Chinese Registered Dietitian** |
| --- | --- |
| Nov 2014 | The Registered Dietitian Accreditation System Establishment project by the Chinese Nutrition Society (CNS) launched |
| May 28, 2016 | The first pilot Registered Dietitian Examination in Shanghai Jiao Tong University School of Medicine conducted |
| Dec 17, 2016 | • Registered Dietitian Committee (RDC) by CNS established  • The first annual meeting of RDC held |
| Dec 30, 2016 | *The* *Interim Provisions on the Level Evaluation System of Registered Dietitians* issued |
| Mar 3, 2017 | *The Implementation Standards of Dietitian Registration Examination* issued |
| Mar 7, 2017 | An examination exemption guideline for grandfather group dietitians released |
| Mar 27, 2017 | Statement of *no short-term training for RD/DTR credentials accepted* announced |
| Apr 23, 2017 | Official website of RD/DTR www.crdietitian.org launched |
| May 24, 2017 | *Registered Dietitian: Practice and Future Trends* Forum in the 13th China Nutrition Science Congress held |
| Jul 9, 2017 | The first national RD examination held in five cities – Beijing, Shanghai, Guangzhou, Changchun, Chengdu |
| Aug 17, 2017 | The first cycle of didactic program application and accreditation started |
| Oct 18–24, 2017 | The RDC representatives  • meeted with dietetic program advisors of New York University and Columbia University  • meeted with The New York City Government Department of Health Division of Nutrition  • visited long term care institutions in Manhattan and Bronx  • attended FNCE 2017 in Chicago |
| Dec 7, 2017 | An examination exemption guideline for dietitians overseas released |
| Dec 8, 2017 | The first cycle of dietetic internship program application and accreditation started |
| Dec 18, 2017 | In the CPPCC auditorium, Beijing  • accreditation ceremony of RD representatives held  • the official logo of RD released  • the first cycle of didactic programs accredited |
| Jan 21, 2018 | The first national DTR examination held in five cities – Beijing, Shanghai, Guangzhou, Changchun, Chengdu |
| Mar 17, 2018 | The second annual meeting of RDC and the first seminar of accredited didactic programs held |
| Apr 19, 2018 | 20 registered dietitians under 35 sponsored by the President’s Fund of CNS participating in the 7th Asian Dietitians Conference with either oral or poster accepted |
| Jul 24, 2018 | The second national RD examination held in five cities – Beijing, Shanghai, Guangzhou, Changchun, Chengdu |
| Aug 3, 2018 | *The Interim Provisions on Continuing Education* *of Registered Dietitians* issued |
| Aug 29–31, 2018 | The 1st *Nutrition Theory Application and Skills Practice* CE program (CNS developed) held |
| Oct 13–15, 2018 | The 2nd *Nutrition Theory Application and Skills Practice* CE program (CNS developed) held |
| Nov 16–18, 2018 | The 3rd *Nutrition Theory Application and Skills Practice* CE program (CNS developed) held |
| Jan 20, 2019 | The second national DTR examination held in five cities – Beijing, Shanghai, Guangzhou, Changchun, Chengdu |
| Mar 6, 2019 | The first cycle dietetic internship programs accredited |
| Mar 22, 2019 | The second cycle of didactic program application and accreditation started |
| Jun 6, 2019 | The second cycle of didactic programs accredited |
| Sep 3, 2019 | *Let RD resident in 55 national Olympic sports teams* project launched |
| Sep 22, 2019 | *Registered Dietitian Development Forum – Practice Skills Advancing to the Rising Future* in the 14th China Nutrition Science Congress and the 11th Asia Pacific Conference on Clinical Nutrition held |
| Oct 28–Nov 1, 2019 | The 1st *Sports Nutrition Advanced Skills* CE program (CNS and the General Administration of Sport of China jointly developed) held |
| Nov 21, 2019 | Official CE application and RD/DTR recertification system launched |
| Nov 23, 2019 | The second seminar of accredited didactic programs held |
| Nov 29–Dec 1, 2019 | The 1st *Nutritional Science Popularization Ability Improvement* CE program (CNS developed) held |
| Dec 6–8, 2019 | The 2nd *Nutritional Science Popularization Ability Improvement* CE program (CNS developed) held |
| May 17, 2020 | Strategic cooperation contract of CNS and Health Human Resources Development Center, Ministry of Health of the People’s Republic of China (HHRDC) signed |
| Jul 1, 2020 | The second cycle of *Let RD resident in 55 national Olympic sports teams* project recruitment launched |
| Aug 7–9, 2020 | The 3rd *Nutritional Science Popularization Ability Improvement* CE program (CNS developed) held |
| Dec 20, 2020 | The first computer-based national RD/DTR examination held in 15 cities (the 6^th^ national exam) |

# *Appendix 4 – Selected characteristics of overseas dietitian registered in China*

| **Variable** |  |
| --- | --- |
| **Level** |  |
| RD | 52 (93%) |
| DTR | 4 (7%) |
| **Gender** |  |
| Female | 51 (91%) |
| Male | 5 (9%) |
| **Country of certification** |  |
| The US | 22 (42%) |
| Canada | 8 (15%) |
| Australia | 15 (29%) |
| Japan | 5 (10%) |
| New Zealand | 1 (2%) |
| The UK | 1 (2%) |
| **Highest degree earned** |  |
| Bachelor’s | 10 (18%) |
| Master’s | 44 (78%) |
| Doctoral | 2 (4%) |
| **Age (y)** |  |
| 25–29 | 14 (27%) |
| 30–34 | 28 (54%) |
| 35–39 | 6 (11%) |
| 40–44 | 3 (6%) |
| >44 | 1 (2%) |
| **Practice area** |  |
| Clinical nutrition | 20 (38%) |
| Consultation and business | 20 (38%) |
| Education and research | 6 (12%) |
| Food and nutrition management | 3 (6%) |
| Community | 3 (6%) |
